# Effect of intermittent fasting on circulating inflammatory markers in obesity: A review of human trials

**DOI:** 10.3389/fnut.2023.1146924

**Published:** 2023-04-17

**Authors:** Andrea Mulas, Sofia Cienfuegos, Mark Ezpeleta, Shuhao Lin, Vasiliki Pavlou, Krista A. Varady

**Affiliations:** Department of Kinesiology and Nutrition, University of Illinois at Chicago, Chicago, IL, United States

**Keywords:** intermittent fasting, time restricted eating, alternate day fasting, inflammation, weight loss, visceral fat, obesity

## Abstract

Obesity is associated with low-grade inflammation. Weight loss, by means of dietary restriction, has been shown to reduce systemic inflammation. Intermittent fasting has recently gained popularity as a weight loss diet, but its effects on inflammatory markers in individuals with obesity have yet to be summarized. Accordingly, this review examined how the two main forms of intermittent fasting, i.e., time restricted eating (TRE) and alternate day fasting (ADF), impact body weight and key circulating inflammatory markers (i.e., C-reactive protein (CRP), tumor necrosis factor-alpha (TNF-alpha), and interleukin-6 (IL-6)), in adults with obesity. Results from this review reveal that TRE with various eating window durations (4–10 h per day) has no effect on circulating levels of CRP, TNF-alpha or IL-6, with 1–5% weight loss. As for ADF, reductions in CRP concentrations were noted when >6% weight loss was achieved. However, ADF had no effect on TNF-alpha or IL-6 concentrations, with this degree of weight loss. Thus, intermittent fasting has little or no effect on key inflammatory markers, but more research is warranted to confirm these preliminary findings.

## 1. Introduction

Inflammation helps an organism to protect itself from harmful stimuli and initiate the healing of damaged cells. Once the initial trigger is eliminated, acute inflammation tends to resolve quickly. However, when the trigger is not eliminated, acute inflammation becomes chronic. Obesity is associated with chronic low-grade inflammation (1). Individuals with obesity display a two to three-fold increase in circulating levels of C-reactive protein (CRP), tumor necrosis factor-alpha (TNF-alpha), and interleukin-6 (IL-6), when compared to their normal weight counterparts (2–4). The key source of inflammation associated with obesity is white adipose tissue. Under metabolic stress, these white adipocytes produce a plethora of inflammatory cytokines and chemoattractant molecules that activate and recruit resident and non-resident immune cells (5). These immune cells, e.g., T-lymphocytes and macrophages, perpetuate the inflammatory state in white adipose tissue. If left unchecked, pro-inflammatory cytokines derived from adipose tissue go on to disrupt insulin signaling, which can lead to the development of insulin resistance, and eventually, type 2 diabetes (6).

Weight loss (5–10% from baseline) by dietary restriction plays a crucial role in lowering pro-inflammatory gene expression and chronic inflammation (7). In recent years, intermittent fasting has gained popularity as a dietary weight loss regimen (8). Two forms of intermittent fasting are time restricted eating (TRE) and alternate day fasting (ADF). TRE involves confining the eating window to a certain number of hours per day (usually 4–10 h) and fasting with energy free beverages for the remaining hours. ADF, on the other hand, involves a “fast day,” where 0–500 kcals are consumed, alternated with a “feast day,” where one is permitted to eat *ad libitum*. These diets produce approximately 1–12% weight loss over 2–12 months. In addition, these protocols have been shown to lower fasting insulin and insulin resistance in adults with obesity (8, 9).

The impact of intermittent fasting on circulating inflammatory cytokine concentrations in adults with obesity remains unclear. While several human studies have examined the effects of TRE (10–14) and ADF (15–20) on pro-inflammatory markers, the results of these trials have yet to be evaluated as a whole. Accordingly, the goal of this narrative review is to summarize the effects of TRE and ADF on body weight and key circulating inflammatory markers, i.e., CRP, TNF-alpha, and IL-6, in adults with overweight and obesity.

## 2. Methods–human trial selection

A PubMed, Embase, and Cochrane Library search was conducted using the following key words: “intermittent fasting,” “fasting,” “intermittent energy restriction,” “intermittent calorie restriction,” “alternate day fasting,” ““time restricted eating,” “time restricted feeding,” “inflammation,” “inflammatory markers,” “weight loss,” “obesity,” “C-reactive protein,” “tumor necrosis factor-alpha,” “interleukin-6,” and “inflammatory cytokines.” Inclusion criteria for research articles were as follows: (a) randomized trials which included a control arm or a comparator arm (e.g., daily energy restriction diets), (b) adult male and/or female participants with overweight or obesity, and (c) end points that included changes in body weight and a relevant inflammatory marker (i.e., CRP, TNF-alpha, or IL-6). The following exclusion criteria were applied: (a) cohort studies, observations studies, and non-randomized trials (b) fasting performed as a religious practice (Islam or Seventh-Day Adventist), (c) trial durations of less than 1 week, (d) trials that provided eucaloric diets during intermittent fasting to prevent weight loss, (e) studies that included only normal weight participants, and (f) studies that included participants diagnosed with type 1 or type 2 diabetes (however participants with prediabetes were included). Our search retrieved five human trials of TRE (Table 1) and six trials of ADF (Table 2). Please note, the study by Cienfuegos et al. (10) has been listed in Table 1 as two separate studies since the trial involved a 4-h TRE and 6-h TRE arm, and we wanted to distinguish the effects of these two unique TRE diets on inflammatory outcomes.

**Table 1:** Effect of time restricted eating on circulating inflammatory markers in adults with obesity.

Reference	Subjects	Length (weeks)	Design and Interventions	Body weight (% change)	Fat mass (% change)	Visceral fat (% change)	hs-CRP (% change)	CRP (% change)	TNF-alpha (% change)	IL-6 (% change)
4-h Time restricted eating										
Cienfuegos 2020 (10)	*n* = 58, MF obese no diabetes	8	RCT: Parallel-arm 1. 4-h TRE (3-7 pm) 2. Control (no meal timing restrictions)	1. ↓3%† 2. ∅	1. ↓6%† 2. ∅	1. ∅ 2. ∅	--	--	1. ∅ 2. ∅	1. ∅ 2. ∅
6-h Time restricted eating										
Cienfuegos 2020 (10)	*n* = 58, MF obese no diabetes	8	RCT: Parallel-arm 1. 6-h TRE (1-7 pm) 2. Control (no meal timing restrictions)	1. ↓ 3%*† 2. ∅	1. ↓3%† 2. ∅	1. ∅ 2. ∅	--	--	1. ∅ 2. ∅	1. ∅ 2. ∅
Zhang 2022 (14)	*n* = 60, MF obese no diabetes	8	RCT: Parallel-arm 1. 6-h TRE (7 am-1 pm) 2. 6-h TRE (12–6 pm) 3. Control (no meal timing restrictions)	1. ↓ 5%*† 2. ↓ 4%*† 3. ∅	1. ↓8%*† 2. ↓9%*† 3. ∅	1. ↓11%*† 2. ↓11%*† 3. ∅	1. ∅ 2. ∅ 3. ∅	--	1. ∅ 2. ∅ 3. ∅	1. ∅ 2. ∅ 3. ∅
8-h Time restricted eating										
Kotarsky 2021 (11)	*n* = 23, MF obese no diabetes	8	RCT: Parallel-arm 1. 8-h TRE (12 pm-8 pm) + Resistance training 3x/week 2. Controls (no meal timing restrictions) + Resistance training 3x/week	1. ↓4%*† 2. ∅	1. ↓9%*† 2. ∅	1. ↓13%* 2. ∅	1. ∅ 2. ∅	--	--	--
Shroder 2021 (13)	*n* = 20, F obese no diabetes	12	RCT: Parallel-arm 1. 8-h TRE (12 pm-8 pm) 2. Control (no meal timing restrictions)	1. ↓4%*† 2. ↑1%*	1. ↓6%*† 2. ∅	--	--	1. ∅ 2. ∅	--	--
10-h Time restricted eating										
Manoogian 2022 (12)	*n* = 150, MF overweight obese no diabetes	12	RT: Parallel-arm 1. 10-h TRE (self-selected) + Mediterranean diet 2. Control (no meal timing restrictions) + Mediterranean diet	1. ↓1%*† 2. ∅	--	--	--	1. ∅ 2. ∅	--	--

CRP: C-reactive protein, hs-CRP: High sensitivity C-reactive protein, F: Female, IL-6: Interleukin-6, M: Male, RCT: Randomized controlled trial, TNF-alpha: Tumor necrosis factor-alpha, TRE: Time restricted eating (prescribed eating window shown in parentheses).

-- Not measured, ∅: No statistically significant change.

* *p* < 0.05, Significantly different from baseline (within group effect).

† *p* < 0.05, Significantly different from the control or comparison group (between group effect).

**Table 2:** Effect of alternate day fasting on circulating inflammatory markers in adults with obesity.

Reference	Subjects	Length(weeks)	Design and Interventions	Body weight(% change)	Fat mass(% change)	Visceral fat(% change)	hs-CRP(% change)	CRP(% change)	TNF-alpha(% change)	IL-6(% change)
Cho 2019 (17)	*n* = 112, MF Overweight obese no diabetes	8	RCT: Parallel-arm 1. ADF Fast day (500 kcal) Feast day (*ad lib*) 2. ADF + Exercise 3. Exercise (endurance) 4. Control (usual diet)	1. ↓5%*† 2. ↓5%*† 3. ↓3%* 4. ∅	1. ↓13%*† 2. ↓12%*† 3. ↓7%* 4. ∅	1. ∅ 2. ∅ 3. ∅ 4. ∅	1. ∅ 2. ∅ 3. ∅ 4. ∅	--	--	--
Castela 2020 (16)	*n* = 28, MF obese no diabetes	12	RT: Parallel-arm 1. ADF Fast day (500 kcal) Feast day (*ad lib*) 2. CR (~1,500 kcal/d)	1. ↓12% * 2. ↓12% *	1. ↓13%* 2. ↓11%*	--	--	--	--	1. ∅ 2. ∅
Varady 2013 (20)	*n* = 32, MF overweight no diabetes	12	RCT: Parallel-arm 1. ADF Fast day (500 kcal) Feast day (*ad lib*) 2. Control (usual diet)	1. ↓6%*† 2. ∅	1. ↓12%*† 2. ∅	--	--	1. ↓13%† 2. ∅	--	--
Razavi 2021 (18)	*n* = 80, MF obesity no diabetes	16	RT: Parallel-arm 1. ADF Fast day (500 kcal) Feast day (*ad lib*) 2. CR (~1,500 kcal/d)	1. ↓7%* 2. ↓5%*	1. ↓15%*† 2. ↓12%*	--	1. ↓48%*† 2. ↓25%*	--	1. ∅ 2. ∅	1. ∅ 2. ∅
Bowen 2018 (15)	*n* = 162, MF overweight obese no diabetes	16	RT: Parallel-arm 1. ADF Fast day (500 kcal) Feast day (*ad lib*) + low carb high protein diet 2. CR (~1,500 kcal/d) + low carb high protein diet	1. ↓10%* 2. ↓12%*	1. ↓18%* 2. ↓22%*	--	1. ↓18%* 2. ↓13%*	--	--	--
Trepanowski 2018 (19)	*n* = 100, MF overweight obese no diabetes	24	RCT: Parallel-arm 1. ADF Fast day (500 kcal) Feast day (*ad lib*) 2. CR (1,500 kcal/d) 3. Control (usual diet)	1. ↓7%*† 2. ↓7%*† 3. ∅	1. ↓12%*† 2. ↓12%*† 3. ∅	1. ↓24%*† 2. ↓29%*† 3. ∅	--	--	1. ∅ 2. ∅ 3. ∅	1. ∅ 2. ∅ 3. ∅

ADF: Alternate day fasting, CR: Calorie restriction, CRP: C-reactive protein, hs-CRP: High sensitivity C-reactive protein, F: Female, IL-6: Interleukin-6, M: Male, RCT: Randomized controlled trial, RT: Randomized trial, TNF-alpha: Tumor necrosis factor-alpha.

-- Not measured, ∅: No statistically significant change.

* *p* < 0.05, Significantly different from baseline (within group effect).

† *p* < 0.05, Significantly different from the control or comparison group (between group effect).

## 3. Effects of intermittent fasting on body weight, fat mass, and visceral fat mass

Obesity is a complex disease associated with an increase in several inflammatory markers, leading to chronic low-grade inflammation (1). In lean healthy individuals, white adipose tissue is confined primarily to subcutaneous depots. However, in people with obesity, white adipocytes can accumulate ectopically within the visceral cavity. The expansion of visceral adipose tissue stimulates the recruitment of macrophages (5, 21). These macrophages assume an inflammatory phenotype and produce cytokines that directly interfere with insulin signaling (5, 21). As such, visceral adipose tissue releases more CRP, TNF-alpha, and IL-6 than subcutaneous adipose tissue (22). Thus, decreasing visceral fat, by means of weight loss, may help to lower systemic inflammation.

### 3.1. Time restricted eating: Body weight

Our search retrieved one study of 4-h TRE (10), two studies of 6-h TRE (10, 14), two studies of 8-h TRE (11, 13), and one study of 10-h TRE (12) (Table 1). The trial populations consisted of men and women with overweight and obesity, but without diabetes. All trials included a control group, where subjects were permitted to eat freely without any meal timing restrictions. Findings from these trials reveal that TRE produces 1–5% weight loss over 8 to 12 weeks, relative to controls (10–14). In these trials, fat mass was reduced by 3–9%, versus controls (10–14). Lean mass, on the other hand, was maintained in each trial (data not shown), relative to controls (10–14). Visceral fat mass was only evaluated in a few studies (10, 11, 14). Visceral fat mass decreased by 11–13% in the trials that produced 4–5% weight loss (11, 14) but remained unchanged in the studies that produced only 3% weight loss (10). Shorter eating windows (4–6 h TRE) (10, 14) did not produce greater reductions in body weight, fat mass, or visceral fat mass, when compared to longer eating windows (8-10-h TRE) (11–13). Interestingly, longer trials (12 weeks) (12, 13) did not produce greater weight loss than shorter trials (8 weeks) (10, 11, 14).

### 3.2. Alternate day fasting: Body weight

We retrieved a total of six ADF human trials that met our inclusion criteria (15–20; Table 2). In all of these trials, subjects were asked to consume 500 kcal (usually as a lunch or dinner) on “fast days” and eat *ad libitum* on alternating “feast days.” The study populations consisted of men and women with overweight and obesity. None of the trials included people with diabetes. The comparator group was either a no-intervention control group, or a daily calorie restriction (CR) group (consuming ~1,500 kcal daily, equivalent to ~25% restriction). After 8–24 weeks of ADF, body weight decreased by 5–12%, relative to baseline (15–20). Reductions in body weight were not statistically different compared to CR (15–19). Fat mass decreased by 12–18% with ADF, while lean mass was maintained (data not shown) (15–20). Visceral fat mass was only assessed in two ADF trials (17, 19). In the study by Cho et al. (17), visceral fat mass did not change after 8 weeks of ADF, versus controls, despite 5% weight loss. Contrary to these findings, Trepanowski et al. (19) reported a 24% decrease in visceral fat mass after 24 weeks of ADF and 7% weight loss, relative to controls. In this study, reductions in visceral fat in the ADF group were similar to that of the CR group (29% decrease in visceral fat, with 7% weight loss) (19).

## 4. Effect of intermittent fasting on circulating CRP concentrations

CRP is an acute-phase protein synthesized by the liver in response to factors released by macrophages and adipocytes (23). Its main role is to tag the surface of dead or dying cells and activate an immune response (24). As part of the immune response, CRP activates the expression of pro-inflammatory cytokines (25). Although this an important physiological process, chronic elevations of CRP have been linked to elevations in insulin resistance and type 2 diabetes risk (26). Therefore, CRP is routinely tested in healthcare settings to measure inflammation caused by chronic disease, infection, or injury. High sensitivity CRP (hs-CRP) is a more sensitive test than the standard CRP test. hs-CRP is able to identify smaller changes in CRP when compared to the regular test. Weight loss has been shown to lower hs-CRP and CRP concentrations in adults with obesity (27). Results from a meta-analysis demonstrate that for each 1 kg of weight loss, the mean concentration of CRP is lowered by 0.13 mg/l (27). Thus, weight loss may be an effective non-pharmacological strategy to lower levels of this pro-inflammatory cytokine.

### 4.1. Time restricted eating: CRP

Changes in hs-CRP or CRP levels were assessed in four TRE studies (11–14) (Table 1). Zhang et al. (14) examined the effect of early 6-h TRE (7 am-1 pm eating window) versus late 6-h TRE (12 pm-6 pm eating window) on hs-CRP concentrations. After 8 weeks, hs-CRP concentrations did not change in either the early or late TRE groups, relative to controls, despite 4–5% weight loss (14). In the study by Kotarsky et al. (11), 8-h TRE (12 pm-8 pm eating window) was combined with resistance training 3 days per week. By the end of the 8-week study, hs-CRP concentrations remained unchanged, relative to controls (11). Shroder et al. (13) also reported no change in CRP concentrations after 12 weeks of 8-h TRE (12 pm-8 pm eating window), versus controls, despite 4% weight loss. Likewise, Manoogian et al. (12) found no significant change in CRP concentrations with 10-h TRE, though this is not surprising, as weight loss in this study was minimal (1%).

### 4.2. Alternate day fasting: CRP

Four ADF trials measured hs-CRP or CRP concentrations (15, 17, 18, 20) (Table 2). Almost all studies reported reductions in hs-CRP or CRP (ranging from 18 to 48%) with 6–10% weight loss (15, 18, 20). The only study that showed no change in CRP was conducted by Cho et al. (17). In this study, body weight decreased by 5% (17). Since the trials that observed significant reductions in CRP all produced greater than 6% weight loss (15, 18, 20), it stands to reason that >6% weight loss may be necessary to see significant modulations in CRP levels with intermittent fasting. This hypothesis warrants confirmation by future trials. The effect of fasting versus daily restriction on CRP levels was also evaluated. In the study by Razavi et al. (18), both the ADF and CR groups lowered hs-CRP concentrations, but the decrease in the ADF group (48%) was significantly greater than that of the CR group (25%). In contrast, Bowen et al. (15) reported reductions in hs-CRP by ADF (18%) and CR (13%), with no differences between groups.

## 5. Effect of intermittent fasting on circulating TNF-alpha concentrations

TNF-alpha is a pro-inflammatory cytokine. The recruitment and activation of immune cells in white adipose tissue systemically augments circulating levels of TNF-alpha (28). Higher plasma concentrations of TNF-alpha have been implicated in the development of insulin resistance (28). More specifically, excessive TNF-alpha promotes skeletal muscle insulin resistance by negatively regulating insulin signal transduction to glucose uptake (29). Reductions in body weight have been shown to lower circulating levels of TNF-alpha in adults with obesity (7, 30). However, accumulating evidence suggests that at least 10% weight loss may be needed to see beneficial reductions in this inflammatory marker (7, 30).

### 5.1. Time restricted eating: TNF-alpha

Changes in TNF-alpha levels during TRE were evaluated in the studies by Cienfuegos et al. (10) and Zhang et al. (14) (Table 1). In the study by Cienfuegos et al. (10), men and women with obesity were randomized to either 4-h TRE (3 pm-7 pm eating window) or 6-h TRE (1 pm-7 pm eating window). After 8 weeks, body weight decreased by 3% in both groups, but TNF-alpha concentrations remained unchanged, versus controls (10). In the trial by Zhang et al. (14), participants with obesity followed either early 6-h TRE (7 am-1 pm eating window) or late 6-h TRE (12 pm-6 pm eating window) for 8 weeks. At the conclusion of the study, body weight decreased in the early TRE group by 5%, and the late TRE group by 4%, but TNF-alpha did not change, versus controls (14).

### 5.2. Alternate day fasting: TNF-alpha

The effect of ADF on TNF-alpha concentrations in adults with obesity was assessed in two trials (18, 19) (Table 2). In the study by Razavi et al. (18), adults with obesity were randomized to either an ADF or CR diet. After 16 weeks of intervention, the ADF and CR groups lost 7 and 5% of body weight, respectively, but TNF-alpha levels remained unchanged, relative to controls (18). Similarly, Romanowski et al. (19) found that 24 weeks of ADF or CR reduced body weight by 7% in both groups, but this degree of weight loss had no effect on TNF-alpha levels in adults with overweight or obesity.

## 6. Effect of intermittent fasting on circulating IL-6 concentrations

IL-6 has is a pro-inflammatory cytokine that induces the development of insulin resistance (31). The synthesis and secretion of IL-6 in visceral fat is almost three times higher than that of subcutaneous fat (32). IL-6 causes insulin resistance by impairing the phosphorylation of the insulin receptor (31). Reductions in body weight and visceral fat mass by diet (33) and bariatric surgery (34) have been shown to reduce circulating levels of IL-6 in individuals with obesity, though findings are variable (35). To date, no weight loss threshold has been established that corresponds to significant decreases in IL-6 concentrations.

### 6.1. Time restricted eating: IL-6

Only two TRE studies have examined changes in IL-6 concentrations with weight loss (Table 1). Cienfuegos et al. (10) observed no changes in circulating IL-6, relative to controls, with either 4-h TRE or 6-h TRE, in men and women with obesity. However, it should be noted that weight loss for both groups was minimal (3%) and no changes in visceral fat mass were observed. Zhang et al. (14) also reported no change in IL-6 levels, versus controls, with either early or late 6-h TRE, even though significant weight loss (4–5%) and visceral fat mass reductions were noted in both intervention groups.

### 6.2. Alternate day fasting: IL-6

Changes in circulating IL-6 concentrations were assessed in three human trials of ADF (Table 2). In the study by Castela et al. (16), adults with obesity followed an ADF or CR protocol for 12 weeks. By the end of the study, body weight decreased by 12% in both groups, but circulating levels of IL-6 remained unchanged (16). Ravazi et al. (18) also demonstrated no change in IL-6 levels after 16 weeks of ADF or CR, despite both groups achieving clinically significant weight loss (5–7%). Likewise, Trepanowski et al. (19) observed no effect on plasma IL-6 after 24 weeks of ADF or CR, versus controls, even though both interventions groups lost 7% of body weight and decreased visceral fat mass by 24–29%.

## 7. Summary of findings

### 7.1. Time restricted eating: Summary

Findings from this review suggest that TRE with various eating window durations (4–10 h) has no effect on circulating levels of CRP, TNF-alpha or IL-6, in adults with overweight or obesity (10–14). However, it is possible that the degree of weight loss and visceral fat mass loss observed in these trials was not high enough to produce favorable modulations in these inflammatory parameters. Indeed, mounting evidence suggests that more than 5% weight loss, or even 10% weight loss, may be necessary to significantly reduce CRP, TNF-alpha, and IL-6 (27, 30, 33, 36, 37). Since these studies only produced body weight reductions ranging from 1 to 5%, this may explain why no beneficial changes in these inflammatory parameters were observed.

### 7.2. Alternate day fasting: Summary

Our results suggest that ADF has no effect on circulating levels of TNF-alpha or IL-6 (16, 18, 19), after 8–24 weeks, in adults with overweight or obesity. However, consistent reductions in CRP concentrations (ranging from 13 to 48%) were noted in the ADF trials that produced at least 6% weight loss (15, 18, 20). Thus, preliminary evidence shows that ADF may be an effective diet therapy to lower CRP in adults with obesity. However, more data from well-powered randomized controlled trials (RCTs) will be required to confirm this finding. The degree of visceral fat mass reduction needed to lower plasma CRP with ADF is still unknown, since none of the studies that observed reductions in CRP measured visceral fat. The effect of ADF versus traditional dieting (daily CR) on CRP levels, was also compared. One study (18) showed a two-fold greater decrease in CRP by ADF versus CR, while another study (15) showed no difference in CRP reduction between diet groups. The reason for these conflicting findings is not certain since ADF and CR produced similar weight loss in these studies. Whether intermittent fasting produces more pronounced decreases in CRP versus traditional dieting warrants further investigation.

## 8. Directions for future research

The evidence in this area is still very limited. It will be of interest for future trials to investigate how intermittent fasting impacts circulating levels inflammatory markers in different population groups, including those with diabetes and other metabolic disorders, such as non-alcoholic fatty liver disease (NAFLD) and polycystic ovary syndrome (PCOS). It will also be important to see how fasting impacts these inflammatory parameters over longer periods of active weight loss (>6 months) and during periods of weight maintenance. Future studies should also measure both gene expression and circulating concentrations in a wider array of cytokines, including monocyte chemoattractant protein-1 (MCP-1), IL-10, leptin, adiponectin, visfatin, and resistin (38–42). Measuring changes in visceral fat mass and subcutaneous fat mass using robust methods (e.g., magnetic resonance imaging (MRI)), and correlating changes in these fat depots with changes in cytokines, should also be prioritized.

## 9. Limitations to the current body of evidence

There are several limitations to the current body of evidence. First, we were only able to retrieve a small amount of intermittent fasting randomized trials (11 total) that assessed both body weight and inflammation in adults with obesity. Second, most studies had small sample sizes and measured inflammatory markers as secondary exploratory outcomes. Thus, it is likely that none of these trials were adequately powered to detect significant changes in inflammation. Third, many trials did not include a control group in their design. Thus, it is difficult to confirm if these results are due to the fasting intervention instead of other extraneous variables. Fourth, most of these trials were short, so the long-term effects of intermittent fasting on inflammatory markers is still not known. Fifth, these studies only examined healthy adults with obesity. It will be of interest to see if these findings hold true in people with diabetes or other obesity-related comorbidities. Lastly, many of the TRE studies did not employ rigorous control groups (i.e., controls were permitted to eat freely instead of being assigned a specific longer eating window).

## 10. Conclusion

Results of this review suggest that intermittent fasting has little or no effect on key circulating pro-inflammatory cytokines. Though ADF decreased plasma CRP when >6% weight loss was achieved, it had no effect on TNF-alpha or IL-6. As for TRE, no changes in any inflammatory parameter (CRP, TNF-alpha, or IL-6) were observed, even with 5% weight loss. Nevertheless, the data in this area are still very limited, and large scale, well-powered RCTs designed to specifically examine how intermittent fasting impacts inflammatory markers, will be needed to confirm these findings.

## Author contributions

All authors listed have made a substantial, direct, and intellectual contribution to the work and approved it for publication.

## Conflict of interest

KV received author fees from Hachette Book Group for the book, “The Every Other Day Diet.”

The remaining authors declare that the research was conducted in the absence of any commercial or financial relationships that could be construed as a potential conflict of interest.

## Publisher’s note

All claims expressed in this article are solely those of the authors and do not necessarily represent those of their affiliated organizations, or those of the publisher, the editors and the reviewers. Any product that may be evaluated in this article, or claim that may be made by its manufacturer, is not guaranteed or endorsed by the publisher.
